# Associations of social and environmental supports with sedentary behavior, light and moderate-to-vigorous physical activity in obese underserved adolescents

**DOI:** 10.1186/s12966-014-0092-1

**Published:** 2014-08-15

**Authors:** Hannah G Lawman, Dawn K Wilson

**Affiliations:** Center for Obesity Research and Education, Temple University, 3223 N. Broad Street suite 175, Philadelphia, PA 19140 USA; Department of Psychology, University of South Carolina, Columbia, SC USA

**Keywords:** Parent support for physical activity, Neighborhood support for physical activity, Self-efficacy for physical activity, Accelerometer, Minority, Overweight, Childhood obesity

## Abstract

**Background:**

Evidence to support differential health impacts of sedentary behavior (SB), light physical activity (LPA), and moderate-to-vigorous physical activity (MVPA) is building. However, few studies have examined individual, social, and environmental supports across the full range of sedentary and physical activities, including key influences such as self-efficacy, parenting factors, and home and neighborhood resources. This may be particularly important in underserved (low-income, minority), overweight/obese adolescents due to the social and environmental challenges (lack of resources, etc.) associated with increasing MVPA. This study evaluated a range of bioecological factors including individual (self-efficacy), parental (parental support, monitoring, limit-setting, and nurturance), and environmental (perceived home resources for PA and neighborhood support for PA) predictors of SB, LPA and MVPA in overweight/obese adolescents.

**Methods:**

Overweight/obese and predominantly minority adolescents and caregivers (n = 181) completed measures in 2010 in the US including surveys assessing self-efficacy for PA, parenting variables related to PA and home and neighborhood supports for PA. Outcomes included 7-day accelerometer estimates of SB, LPA, and MVPA.

**Results:**

Regression analyses showed parental social support and neighborhood support were significantly associated with LPA. No significant associations were found for SB or MVPA.

**Conclusions:**

Results emphasized the importance of examining a range of sedentary and PA intensities and highlighted the role of parental and neighborhood social supports for LPA. These results have important implications that suggest that health promotion efforts should target social and environmental supports for increasing LPA in youth who are overweight/obese.

## Introduction

Increasing evidence suggests that social and physical environments are important determinants of obesity and physical activity (PA) in youth [1,2]. However, only limited research has investigated social and environmental determinants across the full continuum of sedentary and PA intensities (i.e., sedentary (SB), light (LPA), moderate, and vigorous (MVPA)) [3]. This may be particularly important in underserved (low-income, racial/ethnic minority) and overweight/obese youth who display a greater number of cardiometabolic risk factors [4] and face increased barriers for engaging in PA [5].

The current study utilizes the bioecological model [6] to provide a guiding framework for understanding individual, family, and environmental determinants of SB, LPA, and MVPA in youth. Additionally, the EnRG framework adds to the bioecological model by hypothesizing that dual-processes are important in testing whether environmental factors may influence behavior both directly and indirectly [1]. Previous studies have supported perceived and objective environmental factors as moderators of the link between individual cognitive factors and behavior [7,8]. Consistent with the bioecological model and the EnRG framework’s emphasis on cognitive factors such as perceived behavioral control, one potentially important individual level factor is self-efficacy. Previous reviews [9], meta-analyses [10], and studies with underserved youth [11] have also shown that self-efficacy is a key cognitive factor in understanding youth’s MVPA. At the environmental level, key factors in understanding youth MVPA have included home and neighborhood characteristics such availability of resources, safety, and neighborhood social support [12,13]. However, this study expands on previous research by hypothesizing that parenting variables including parent support [14], monitoring and limit-setting [15-17], and parental nurturance [18] will be important determinants of SB and PA in youth beyond environmental factors.

The influence of social environmental and parenting factors may also differ as a function of PA intensity [19]. Although few investigators have evaluated a broader continuum of sedentary and PA intensity outcomes, evidence is building supporting health benefits of engaging in LPA and limiting SB [20-25]. LPA has been defined as energy expenditure at the level of 1.6-2.9 metabolic equivalents (e.g., slow walking, sitting and writing, cooking, washing dishes), increases metabolic rate, and contributes to total daily energy expenditure [26]. Targeting increasing LPA as a way to decrease SB may be a unique intervention strategy in high-risk youth (minority, overweight/obese) and offers a number of potential benefits compared to targeting MVPA. LPA may be easier to increase given its higher occurrence and ability to address common barriers of MVPA such as injury, safety, cost and access concerns and feeling embarrassed of low skill levels or sweating [27-31]. Overweight/obese youth may also benefit more from targeting increases in LPA compared to healthy-weight individuals due to the relative increased energy expenditure from lighter activities [32].

Despite the health benefits and potential intervention opportunities of LPA, many studies, including national surveillance studies, do not measure LPA or sedentary behavior (SB) [33,34]. While studies have shown that health behaviors cluster in consistent and inconsistent ways [35], previous literature has tended to emphasize only one behavior within the SB and PA spectrums [3,36]. Thus, although a substantial amount of literature has investigated self-efficacy, parenting factors, and home and neighborhoods supports in MVPA, findings remain mixed [10,35], and literature examining these key factors in relation to SB or LPA is lacking. Therefore, the current study expands on previous studies by investigating self-efficacy, parenting, and perceptions of social environmental factors across all activity intensities, including SB, LPA, and MVPA.

Considering multiple systems simultaneously (e.g., self-efficacy, parenting, and social environmental factors) can facilitate a broader, contextual, and more complete understanding of the multiple determinants of SB, LPA, and MVPA. However, a recent review showed studies on family and environmental correlates of SB and MVPA, specifically in underserved youth, are inconsistent and seldom investigate influences from multiple levels [28]. Thus, the purpose of the current study was to examine associations of individual (i.e., self-efficacy), social (i.e., parental support, nurturance, limit-setting, and monitoring), and environmental (perceptions of home resources and neighborhood supports for PA) factors with SB, LPA and MVPA in overweight/obese, underserved adolescents.

## Methods

### Participants and procedure

Participants were 201 low-income or racial/ethnic minority adolescents (10–17 years old). Inclusion criteria were BMI at or above the 85th percentile and ability to speak/write English. Exclusion criteria included having a chronic medical condition, developmental disability, or serious psychiatric disorder. The current study targeted overweight, underserved youth in pediatric clinics serving a low-income population (75% Medicaid patients) as this unique group of youth can be considered to be at especially high risk for negative health trajectories. Recruitment efforts included referral through clinic pediatricians, community events, and passive consent (i.e., direct mailing by pediatricians in accordance with HIPAA requirements). Participants were primarily recruited through pediatrician referrals (45%) and passive consent (32%). Participants recruited through pediatricians and passive consent had a significantly higher BMI compared to participants recruited in community events. No differences in SB and MVPA levels were found across recruitment methods. Of those eligible and invited to participate, 45.7% were enrolled, 22.3% declined, 23.0% did not show up for their appointment, and 9% failed to meet BMI inclusion criteria at the first appointment.

Participants took part in two appointments one week apart after completing informed consent. The first visit included consent/assent and anthropometric measures (height, weight, and waist circumference for both parent and child). Directions for wearing accelerometers were given. At the second appointment, accelerometers were collected and questionnaires were administered to participants and caregivers. Families were given educational materials, health information, and $30 compensation for participation. Of 201 families enrolled, 98% returned for the second visit. The study was approved by the institutional review board of the University of South Carolina.

### Measures

#### Self-efficacy

The Relapse Prevention factor of a previously validated scale [37] was used in the current study to evaluate barriers for PA. The scale has demonstrated good internal consistency and validity in underserved youth [38]. Adolescents responded to 9 items on PA indicating how confident they were they could motivate themselves to change their exercise habits when facing barriers. This subscale was chosen as high-risk youth may face additional barriers to increasing PA [5]. Responses were recorded on a three-point scale (a little sure, sure, very sure). Internal consistency for the current study was high (α = .87 for PA).

#### Parent social support

Adolescents completed a measure of parent social support for PA using a modified version of a previously validated scale [39]. Participants responded to 13 items indicating how often in the past month a parent has provided support (5 point scale from never to always). Items were averaged to create a social support measure. Previous research has demonstrated acceptable psychometric properties, and a subscale has been shown to be predictive of MVPA in underserved adolescents [39,40]. Internal consistency for the current study was high (α = .89).

#### Parent limit-setting and monitoring

Limit-setting and monitoring subscales of a previously validated scale [41] were used to assess frequency of limits on adolescents’ health behaviors (ie., limiting screen time, sodas, and snacks and tracking of adolescents’ eating and activity habits). Previous research supports behavior-specific measures over general measures [42]. The scales in the current study were chosen to balance a focus on obesity-related health behaviors with participant burden from multiple behavior-specific measures. Parents reported on six questions (strongly disagree to strongly agree) for limit-setting and seven items (never to almost always) for monitoring on five-point scales. Previous literature has supported reliability, factor structure, and predictive validity in parents of minority youth [41]. Internal consistency for the current study was high (α = .86 for both).

#### Parental nurturance

The nurturance subscale of a parenting dimensions scale [18] was used to assess parental warmth and interactive style (e.g., “Family members easily express warmth and caring towards each other”). Parents responded to five items on a five-point scale (not at all to exactly like me). Previous literature has supported the scale’s factor structure, reliability, and stability over time [18]. Internal consistency for the current study was high (α = .82).

#### Home resources

Home resources for PA was parent-reported using 12 items that assessed the availability of PA equipment in the home (e.g., balls, covered areas) and has shown acceptable reliability in an underserved sample [12]. Items were summed, and internal consistency for the current study was marginal (α = .61).

#### Perceived neighborhood support

Parents completed a 16-item measure of perceived neighborhood supports (i.e., within 0.5-mile radius or 10-minute walk) for PA used in previous work with underserved families. The scale has shown acceptable reliability and predictive validity [associated with MVPA; 12]. The scale assessed perceptions of social and physical environmental neighborhood supports for PA (e.g., sidewalks, free from unattended dogs, recreation center, waving to neighbors) using a 5-point scale (strongly disagree to strongly agree). Items were averaged, and internal consistency for the current study was moderate (α = .73).

#### Physical activity

Objective assessments of adolescents’ SB, LPA, and MVPA were obtained with omni-directional accelerometers over seven consecutive days. Each day of accelerometer data was divided into five intervals: 6–9 am, 9–2 pm, 2–5 pm, 5–8 pm, and 8 pm to midnight [43]. Data were recorded in 1-minute epochs [44], and 90 consecutive zero-counts were used to indicate non-wear [45]. Raw activity data were converted into time spent in SB, LPA, and MVPA based on validated Actical-specific activity count thresholds for children identified using a range of sedentary and active tasks by Puyau et al. [46] (where SB: <100, LPA: 100- <1,500; MPA: 1,500- < 6,500, and VPA: >6,500). After imputation (described below) intervals were summed for all time points each day and averaged across days to provide one measure of average daily minutes of SB, LPA, and MVPA, respectively.

### Data analytic plan

Descriptive statistics were examined for demographic characteristics. Analyses by weight status category were not examined as only 2.8% of youth had a BMI percentile ≥ 85th and < 95th [47]. To examine social and environmental associations with SB and PA, three separate multiple regression analyses (SB, LPA, MVPA) were conducted. Independent variables included self-efficacy, parental limit-setting, monitoring, nurturance, parental support, home resources, and neighborhood support. Sex and age were included as covariates. Variables were entered in a stepwise manner to determine the unique variance accounted for by each bioecological level (ie, covariates, individual, social, environmental). Post-hoc power simulations (10,000 simulations, p = .05, n = 201) confirmed the study was 79% powered to detect standardized beta effect sizes of .20.

Participants with no accelerometer data (n = 20) due to lost belts, malfunctions, or less than the 4 days (with ≥10 hours of wear) of valid wear time needed to obtain reliable PA estimates were excluded from analyses [48]. These participants showed no differences from included participants on any study variables. Remaining missing data were dealt with using multiple imputation (20 imputations) to provide unbiased parameter estimates and standard errors as previously proposed [43]. Multiple imputations were conducted in R using the MICE package and included all demographic, psychosocial, SB and PA variables. Seventy-six percent of participants had 7 days of wear, and the remaining participants had some non-compliance but at least 4 days of valid wear. Some surveys or items were missing (~5%). Fractions of missing information are reported.

## Results

### Demographics

Table 1 shows the demographic data for the study sample. Youth were 13.3 ± 2.1 years old, predominantly racial/ethnic minorities (88% minority including 79% African American) and had an average BMI of 33.5 ± 7.0 (see Table 1). Caregivers were 42.2 ± 10.4 years old and had average BMI of 36.9 ± 9.9. Approximately 59% of the sample reported income under $25,000 USD.

**Table 1:** **Participant demographics and psychosocial characteristics (n = 181)**

**Variable**	**Mean**	**Standard deviation**
Female (count/%)	109	60%
Ethnicity (count/%)		
African American	143	79%
Caucasian	21	12%
Latino	15	8%
Other	2	1%
Age (yrs)	13.3	2.1
BMI (kg/m^2^)	33.5	7.0
MVPA (average min/day)	27.6	21.2
Light PA (average min/day)	237.0	71.4
Sedentary behavior (average min/day)	749.0	123.0
Self-efficacy for PA	2.0	0.5
Parental nurturance	4.7	0.9
Parental support	2.6	1.1
Parental limit-setting	3.8	1.0
Parental monitoring	3.5	0.9
Home resources	5.4	2.4
Neighborhood support	2.7	0.5

Note: Values reported using a single imputation and shown as Mean (SD) or Count (%). Self-efficacy was reported using a 3-point scale. Parenting and neighborhood variables were reported using 5-point scales. Home resources were summed and ranged from 0–12. For all scales, higher scores indicated more of the construct. Environmental measures were parent-reported perceptions of home resources and neighborhood supports.

### Correlations

Correlations are shown in Table 2. MVPA was significantly correlated with LPA (r = 0.38) and SB (r = −.34, p < .05 for all). Increased age was significantly associated with higher SB (r = .14) and lower LPA (r = −.34). Higher levels of LPA were associated with higher levels of parental limit-setting (r = .16) and parental monitoring (r = .15, p < .05 for all). SB and MVPA were not significantly associated with any of the individual, social, or environmental factors. Correlations among social factors (r’s ranging from .18 - .58, p < .05 for all) showed parental support, nurturance, limit-setting, and monitoring tended to relate to one another. Home resources and neighborhood supports were also related (r = .19, p < .05).

**Table 2:** **Correlations among individual, social, and environmental determinants and sedentary behavior and physical activity**

	**Age**	**BMI**	**MVPA**	**LPA**	**SB**	**SE**	**PS**	**N**	**LS**	**M**	**HR**
BMI	0.27*										
MVPA	−0.13	−0.09									
LPA	−0.34*	−0.11	0.38*								
SB	0.14*	0.02	−0.34*	−0.08							
Self efficacy	−0.04	−0.07	0.06	0.04	−0.12						
Parental support	−0.07	0.15*	0.09	0.18*	−0.12	0.34*					
Nurturance	−0.11	0.04	0.11	0.01	−0.02	0.09	0.25*				
Limit-setting	−0.29*	−0.08	0.11	0.16*	−0.04	0.07	0.24*	0.24*			
Monitoring	−0.21*	0.03	0.07	0.15*	0.04	0.11	0.18*	0.30*	0.58*		
Home resources	−0.15	−0.12	0.09	−0.01	0.10	−0.11	0.09	0.11	0.14	0.10	
Neighborhood supports	0.01	0.03	0.08	0.13	0.00	0.03	0.04	0.16*	−0.01	−0.01	0.19*

*= p < .05. MVPA = moderate-to-vigorous physical activity, LPA = light physical activity, SB = sedentary behavior.

Notes: Column headings correspond to row names. Environmental measures were parent-reported perceptions of home resources and neighborhood supports.

### Self-efficacy, social and environmental supports and MVPA and LPA

Regression analyses showed that being female was associated with significantly lower levels of MVPA (see Table 3). No other factors were significant. The overall model accounted for 9% of the variance in MVPA. Covariates accounted for 7% of the variance (F(2,178) = 6.75, p < .05), the individual level predictor (self-efficacy) accounted for an additional 0.4% (F(3,177) = 4.72, p < .05), family level predictors (parent support, tracking, limit-setting, nurturance) accounted for an additional 1.2% (F(7,173) = 2.27,<.05), and environmental level predictors (home resources, neighborhood support) accounted for an additional 0.8% (F(9,171) = 1.97, p < .05).

**Table 3:** **Regression analyses predicting adolescents’ moderate-to-vigorous physical activity and light physical activity (n = 181)**

	**Est**	**SE**	**β**	**p**	**Lower CI**	**Upper CI**	**FMI**	**Δ R** ^**2**^
MVPA: (F(9,171) = 1.97, R^2=^.09)								
(Intercept)	19.22	18.56	0.00	0.30	−17.16	55.60	0.06	Covariates = .07
Female	−9.04	3.22	−0.21	<0.01	−15.34	−2.74	0.03	
Age	−0.76	0.80	−0.08	0.34	−2.33	0.80	0.06	
Self-efficacy	1.44	3.25	0.04	0.66	−4.93	7.80	0.05	Individual = .004
Parent support	0.82	1.85	0.04	0.66	−2.81	4.46	0.06	Social = .012
Nurturance	0.44	1.85	0.02	0.81	−3.19	4.07	0.08	
Limit-setting	0.99	2.00	0.05	0.62	−2.93	4.91	0.06	
Monitoring	0.90	2.33	0.04	0.70	−3.67	5.47	0.10	
Home resources	0.13	0.67	0.01	0.85	−1.19	1.45	0.05	Environmental = .008
Neighborhood support	3.47	3.16	0.09	0.27	−2.71	9.66	0.04	
Light PA: (F(9,171) = 5.23, R^2=^.22)								
(Intercept)	350.23	58.42	0.00	<0.01	235.71	464.75	0.04	Covariates = .143
Female	−17.39	10.48	−0.12	0.10	−37.95	3.16	0.07	
Age	−11.33	2.51	−0.33	<0.01	−16.25	−6.40	0.03	
Self-efficacy	−9.47	10.31	−0.07	0.36	−29.69	10.74	0.04	Individual = .001
Parent support	15.67	5.88	0.21	0.01	4.14	27.21	0.05	Social = .045
Nurturance	−8.66	5.85	−0.11	0.14	−20.13	2.81	0.06	
Limit-setting	2.30	6.36	0.03	0.72	−10.17	14.77	0.06	
Monitoring	7.24	7.36	0.09	0.33	−7.19	21.66	0.08	
Home resources	−3.47	2.14	−0.12	0.10	−7.66	0.71	0.04	Environmental = .027
Neighborhood support	19.97	10.15	0.14	0.049	0.08	39.87	0.06	

Est = Estimate, SE = standard error, β = standardized beta, CI = confidence interval, FMI = fraction of missing information.

Note: environmental measures were parent-reported perceptions of home resources and neighborhood supports.

Regression analyses showed that older age was significantly associated with less LPA, and parent social support and neighborhood support were significantly positively associated with LPA (see Table 3). The overall model accounted for 22% of the variance in LPA. Covariates accounted for 14.3% of the variance (F(2,178) = 14.9, p < .05), the individual level predictor (self-efficacy) accounted for an additional 0.1% (F(4,196) = 2.64, p < .05), family level predictors (parent support, tracking, limit-setting, nurturance) accounted for an additional 4.5% (F(7,173) = 5.77, p < .05), and environmental level predictors (home resources, neighborhood support) accounted for an additional 2.7% (F(9,171) = 5.23, p < .05).

### Self-efficacy, social and environmental supports and SB

Regression analyses showed that increased age displayed a trend with increased levels of SB (see Table 4). No other factors were significant. The overall model accounted for 7.7% of the variance in SB. Covariates accounted for 2.9% of the variance (F(2,178) = 2.66, p > .05), the individual level predictor (self-efficacy) accounted for an additional 1.4% (F(3,177) = 2.66, p < .05), family level predictors (parent support, tracking, limit-setting, nurturance) accounted for an additional 1.7% (F(7,173) = 1.57,>.05), and environmental level predictors (home resources, neighborhood support) accounted for an additional 1.7% (F(9,171) = 1.59, p > .05).

**Table 4:** **Regression analyses predicting adolescents’ sedentary behavior (n = 181)**

	**Est**	**SE**	**β**	**p**	**Lower CI**	**Upper CI**	**FMI**	**Δ R** ^**2**^
Sedentary Behavior: (F(9,171) = 1.59, R^2=^.08)								
(Intercept)	621.30	107.25	0.00	0.00	411.09	831.52	0.00	Covariates = .029
Female	26.43	18.95	0.11	0.16	−10.72	63.58	0.00	
Age	8.58	4.61	0.15	0.06	−0.46	17.62	0.00	
Self-efficacy	−17.92	18.99	−0.07	0.35	−55.13	19.30	0.01	Individual = .014
Parent support	−13.48	10.78	−0.10	0.21	−34.61	7.66	0.01	Social = .017
Nurturance	1.72	10.67	0.01	0.87	−19.20	22.64	0.01	
Limit-setting	−6.23	11.57	−0.05	0.59	−28.90	16.44	0.00	
Monitoring	16.59	13.24	0.12	0.21	−9.36	42.54	0.00	
Home resources	7.01	3.92	0.14	0.07	−0.69	14.70	0.00	Environmental = .017
Neighborhood support	−5.77	18.47	−0.02	0.76	−41.97	30.43	0.00	

Est = Estimate, SE = standard error, β = standardized beta, CI = confidence interval, FMI = fraction of missing information.

Note: environmental measures were parent-reported perceptions of home resources and neighborhood supports.

## Discussion

The current study simultaneously examined key individual, social and perceived environmental predictors of accelerometer measured SB, LPA and MVPA in youth who were at high-risk for later chronic health diseases. Results of the current study showed parent social support and perceived neighborhood support (including safety, access, aesthetics, and social cohesion) were significantly positively associated with LPA. However, no significant associations with SB or MVPA were observed.

Results from the current study are among the first to demonstrate that parental social support and perceived neighborhood support (including safety, access, aesthetics, and social cohesion) are significantly associated with accelerometer-measured LPA in overweight/obese youth. In addition, family and environmental levels accounted for more variance in MVPA and LPA than the individual level. In line with the dual-process view of direct environmental influences, this may indicate that parental and neighborhood supports cue some automatic and habitual processes that regulate LPA [1]. Previous literature investigating individual and environmental factors has explored the role of environmental access in facilitating intentions and personality factors for MVPA [7,8]. The current study expands on previous work through the inclusion of parenting factors, which may be particularly important for high-risk youth. While previous studies examining parenting variables for youth’s MVPA have shown inconsistent findings [49], the current study expands on past work by showing that both parental and perceived social environmental supports are associated with higher levels of LPA but not SB or MVPA in overweight/obese youth.

Given the growing evidence supporting the health benefits of LPA [3,26], results of the current study support the need for additional research into the key determinants of LPA so that effective interventions targeting LPA may be developed. Previous interventions have utilized strategies, such as promoting dance or other extracurricular physical activities, to simultaneously increase MVPA and decrease SB [50,51] and have also focused on targeting higher intensity PA [52]. Results of the current study may compliment previous intervention strategies [51,53] through helping parents to build support for lower intensity PA in youth and to think creatively about how their neighborhood environment may be used for increasing LPA. For example, interventionists may consider noting the more enjoyable qualities of engaging in LPA for overweight/obese youth that relate to standing and light activities.

In the current study underserved adolescents showed particularly high levels of SB and particularly low levels of LPA and MVPA. Other samples of African American youth have found comparable MVPA levels [27,34,54] but higher levels of LPA [27,55] compared to the current study sample. In addition, the current study found MVPA was significantly positively associated with LPA and negatively associated with SB. Previous research has shown mixed findings with some showing a positive relation with SB and MVPA and others showing a negative relation [35]. In line with the current study’s findings, longitudinal studies with underserved youth have also shown LPA is displaced by sedentary behavior as children age [55,56]. LPA may be important to target because it occurs more frequently and could be more easily modified in this high-risk group. LPA may offer benefits particularly important for high-risk youth who may have more common barriers to MVPA [27-31]. High-risk youth may also benefit from relative increases in energy expenditure from lighter activities compared to healthy-weight youth [32] and may not find LPA to have as great a decrease in reinforcing value compared to MVPA [57]. As such, parental factors and perceptions of social environmental influences on LPA may be distinct from MVPA. Future research should measure independent and joint contributions of key influences across SB, LPA and MVPA to identify the most effective strategies to target behaviors that cluster in healthy ways [35].

The current study also did not show any significant associations with SB or MVPA. Although this was surprising, these findings are similar to others that have demonstrated null or mixed associations between PA and self-efficacy [10], parental support [9], and perceived and actual neighborhood supports [10], even in underserved youth [28]. Similarly, mixed associations have been found for correlates of SB, though they overwhelmingly focus on the specific SB of television viewing [36]. Overall, these studies and the current study indicate that there are a number of complex bioecological influences and that isolated factors can be expected to have modest effects [10]. In the current study it may be that for overweight, underserved youth, parental and neighborhood supports are directly associated with LPA. In contrast, potential moderators or mechanisms of key bioecological factors for MVPA and SB may need to be explored in this unique, high-risk population, such as those proposed by the dual-process model [1]. Future research should target parenting strategies (e.g., autonomy support, norms, attitudes, access), in interventions to increase PA in high-risk youth [53].

There are several limitations with the present study. This study utilized a relatively small sample from a specific demographic of low-income, overweight, minority youth, which limits the generalizability of the findings. The current study used a cross-sectional design and thus cannot be used to draw causal conclusions. Furthermore, measures were not obtained specific to SB versus LPA versus MVPA. In addition, the current study was not powered to examine interactions across bioecological levels or dual-process influences on SB and PA. Future investigations of moderation and mediation employing longitudinal designs and larger samples sizes are needed to better understand how to target interventions for youth who are overweight. For example, dual-processes models propose that cognitive factors will mediate the role of the environment on behavioral outcomes of SB, LPA, and MVPA [1]. However, the study has important strengths including a unique, high-risk sample of underserved youth, the use of accelerometer estimates of SB and PA, and the simultaneous examination of multiple ecological predictors across the full range of SB and PA intensities.

## Conclusions

Overall, results from the current study emphasize the importance of family level and neighborhood level factors on understanding LPA in overweight underserved youth. Specifically, parent support and perceived neighborhood support were associated with higher levels of LPA in high-risk youth but not with SB or MVPA. Future studies should also explore interactions and mediation effects of cognitive and environmental factors of LPA, particularly for high-risk, underserved youth. These results have important implications that suggest that health promotion efforts should target social and environmental supports for increasing LPA in youth who are overweight/obese.

## Abbreviations

SB: Sedentary behavior
PA: Physical activity
LPA: Light physical activity
MVPA: Moderate-to-vigorous physical activity
